# Designing a Text Messaging Intervention to Improve Physical Activity Behavior Among Low-Income Latino Patients With Diabetes: A Discrete-Choice Experiment, Los Angeles, 2014–2015

**DOI:** 10.5888/pcd13.160035

**Published:** 2016-12-22

**Authors:** Magaly Ramirez, Shinyi Wu, Elizabeth Beale

**Affiliations:** 1 Fielding School of Public Health, University of California, Los Angeles, Los Angeles, California; 2 School of Social Work and Viterbi School of Engineering, University of Southern California, Los Angeles, California; 3 Division of Endocrinology, Diabetes and Metabolism, Keck School of Medicine, University of Southern California, Los Angeles, California. Dr Ramirez was affiliated with the Viterbi School of Engineering, University of Southern California, Los Angeles, California, when the research was conducted.

## Abstract

**Introduction:**

Automated text messaging can deliver self-management education to activate self-care behaviors among people with diabetes. We demonstrated how a discrete-choice experiment was used to determine the features of a text-messaging intervention that are important to urban, low-income Latino patients with diabetes and that could support improvement in their physical activity behavior.

**Methods:**

In a discrete-choice experiment from December 2014 through August 2015 we conducted a survey to elicit information on patient preferences for 5 features of a text-messaging intervention. We described 2 hypothetical interventions and in 7 pairwise comparisons asked respondents to indicate which they preferred. Respondents (n = 125) were recruited in person from a diabetes management program of a safety-net ambulatory care clinic in Los Angeles; clinicians referred patients to the research assistant after routine clinic visits. Data were analyzed by using conditional logistic regression.

**Results:**

We found 2 intervention features that were considered by the survey respondents to be important: 1) the frequency of text messaging and 2) physical activity behavior-change education (the former being more important than the latter). Physical activity goal setting, feedback on physical activity performance, and social support were not significantly important.

**Conclusion:**

A discrete-choice experiment is a feasible way to elicit information on patient preferences for a text-messaging intervention designed to support behavior change. However, discrepancies may exist between patients’ stated preferences and their actual behavior. Future research should validate and expand our findings.

## Introduction

Latinos are less likely than non-Latino whites to receive diabetes self-management education (DSME), and they report worse diabetes self-care behaviors (1). Many barriers preclude Latinos from receiving DSME, including health system factors (eg, lack of access to care and provision of health education); provider factors (eg, language and cultural differences, ineffective interpersonal communication); and patient factors (eg, low health literacy and numeracy, cultural differences in health perceptions) (2–5). Readily available communication technologies, such as automated text messaging, are alternative methods for delivering DSME with the potential to reach, engage, and activate self-care behaviors among Latinos. Technologies can use patients’ preferred language, create content targeted to patients’ cognitive abilities, address patients’ needs according to patients’ preferences, and deliver messages at a relatively low cost. A systematic review found that text message interventions (TMIs) delivering health education made significant improvements in glycemic control among people with type 2 diabetes (6); however, most of the studies examined in the review were conducted in Asia, limiting the generalizability of their findings.

Research is needed to design, implement, and evaluate the effectiveness of using technologies such as text messaging to provide health education to vulnerable racial and ethnic minorities and prompt their self-care behaviors (1,7). Evidence on the design of such technologies is insufficient. Although partial guidance is sometimes drawn from behavior-change theories, designing TMIs based on behavior-change theories alone is not sufficient to overcome a major challenge in TMI research: lack of sustained patient engagement (8). Research suggests that people are more likely to use technologies that address their needs and preferences than those that do not (9). Therefore, a sensible approach to designing effective and engaging TMIs may be to incorporate both behavior-change theory and patient preferences. 

Research is lacking on patient preferences for TMIs and how to elicit information about such preferences. One study (10) used a survey to investigate preferences for diabetes self-management support but only assessed preferences for various delivery modes. Another study (11) surveyed young adults to explore their opinions on the attributes of a mobile health application to promote physical activity; in this study, however, participants were Dutch, and most were female, highly educated, and physically active, making the results difficult to generalize. During the development of a TMI for weight loss, another study (12) conducted a focus group to assess, among other things, preferences for type and frequency of messaging, but details on preferences were not provided.

A drawback to the traditional surveys and focus groups used by previous studies is that they cannot quantify degrees of importance of technology intervention features. Additionally, research participants tend to state that all features are equally important; research participants also perceive features evaluated in isolation differently than they perceive features combined in actual products and services (13).

A discrete-choice experiment (DCE) (14) is an innovative, efficient approach that can overcome the limitations of approaches traditionally used to investigate individual preferences for technology-based health self-management interventions. DCEs originated in marketing research but are increasingly used to elicit data on patient preferences for health service delivery (15).

In a DCE, a product or service is described by its attributes. Each attribute has various levels. For example, 1 attribute of a health care delivery system is appointment waiting time; this attribute could have levels of 3 to 6 days, 7 to 10 days, or 11 to 14 days (15). A survey is used to ask participants to state their preferences for hypothetical alternatives of the product or service. Alternatives are described by attributes and differ by attribute levels. Thus, each alternative is a different combination of attribute levels. Responses are used to determine whether the attributes significantly influence preferences, the relative importance of the attributes, and which attribute levels are preferred.

We conducted a study to demonstrate the potential of DCEs to elicit information on the preferences of patients for various TMI attributes. We focused on TMIs that may help to reduce disparities in the receipt of DSME and engagement in self-care behaviors among urban, low-income Latino adults with diabetes. We also focused on TMIs that support changes in physical activity behavior, because people with type 2 diabetes who exercise regularly improve control of blood glucose and insulin sensitivity (16). Only 28% of Latinos with diabetes are sufficiently active (17). Latinos perceive physical activity as one of the most difficult aspects of diabetes self-care, and most DSME delivered via text messaging targets other self-care behaviors (6,18). The primary objective of this study was to demonstrate how a DCE could be used to determine, from the patient perspective, the importance of TMI features in supporting physical activity behavior change. The secondary objective was to demonstrate how a DCE could be used to investigate how feature preferences vary by patient characteristics (eg, age, sex, education).

## Methods

This study was conducted from December 2014 through August 2015. The DCE comprised 4 steps: 1) identifying levels of attributes of the intervention, 2) constructing choice sets and designing a survey, 3) conducting the survey among members of the target audience to measure preferences, and 4) analyzing the data. A convenience sample of 125 survey participants was recruited from an ambulatory care clinic of the Los Angeles County Department of Health Services, a public safety-net health system. The Health Sciences Institutional Review Board at the University of Southern California approved the study.

### Step 1: Identify levels of attributes of the intervention

We derived attributes from 26 behavior-change strategies used in similar interventions (19) and constructed a subset of 12 attributes by excluding those that did not meet at least 2 of 3 criteria: 1) the attribute was linked to a theoretical framework, 2) evidence showed that the attribute could improve physical activity behavior, and 3) the attribute could address physical activity barriers among Latinos.

Because DCE guidelines suggest using 6 or fewer attributes (20), the 12 attributes were combined or re-expressed as 4 attributes: 1) setting physical activity goals, 2) feedback on physical activity performance, 3) education on physical activity behavior change, and 4) social support. We added a fifth attribute, frequency of messaging, because we hypothesized that message frequency would be important to the target audience.

To minimize the cognitive burden on survey respondents, we assigned 2 levels per attribute. DCE guidelines suggest using 2 to 5 levels (20). To comply with guidelines suggesting that levels cover the full range of product and service possibilities, we reviewed published studies and consulted experts to understand how the 5 attributes are typically operationalized and selected the 2 most salient ways as the 2 levels. We finalized attribute and level descriptions (Table 1) after adjusting them for clarity and concision according to feedback obtained during a pilot test involving 6 people (research assistants and clinicians) who work with our target population.

**Table 1:** Attributes and Levels Included in a Survey in a Discrete-Choice Experiment Conducted to Determine Preferences for a Text-Messaging Intervention Designed to Increase Physical Activity Among Low-Income Latino Adults With Diabetes in Los Angeles, California, 2014–2015

Attribute	Level 1	Level 2
Physical activity goal setting	Patient’s doctor recommends physical activity goals	Patient selects his or her own personalized physical activity goals
Feedback on physical activity performance	Patient receives feedback on his or her individual performance	Patient’s performance is compared with that of other patients
Physical activity behavior-change education	Patient’s doctor recommends the educational content	Patient specifies the type of educational content he or she wants to receive
Social support	Family members learn how to offer support	Patient meets other patients so they can support one another
Frequency of messaging	Patient’s doctor recommends how often patient should receive messages	Patient specifies how often he or she wants to receive messages

### Step 2: Construct choice sets and design survey

To construct the choice sets (generally 2 or more hypothetical product or service alternatives), we first generated an experimental design to specify the attribute-level combinations (ie, alternatives) respondents would evaluate in the survey. A full-factorial design would require respondents to evaluate 32 alternatives (5 attributes at 2 levels each). To make the survey more manageable, DCE macros available in SAS software (SAS Institute Inc) were used to construct a D-efficient experimental design that would require respondents to evaluate fewer combinations while minimizing variances of the parameter estimates (21). The resulting experimental design consisted of 12 combinations.

Next, the DCE macros were used to place the 12 alternatives into pairs in a way that would allow us to estimate all parameters (21). Each pair represented a choice set for respondents to evaluate. For the pilot test, we developed several surveys, each with a different number of choice sets generated by SAS. We asked the 6 pilot-test respondents to indicate at what point they felt too burdened to continue evaluating choice sets and determined that respondents could reasonably be expected to evaluate up to 7 choice sets.

On the basis of the pilot findings, we designed a survey with 7 questions that corresponded to the 7 choice sets (Appendix). For each of the 7 survey questions, we designed 2 cards, one for each alternative (Program A and Program B). Each card depicted a man or woman (depending on the respondent’s sex) and was available in English and Spanish. Each card (Figure) had 5 sections that used pictures and text to describe the attributes. Each card included all 5 attributes, but the attribute levels differed from card to card. The attribute-level combinations depicted in each card corresponded directly to the 12 alternatives. 

**Figure:**
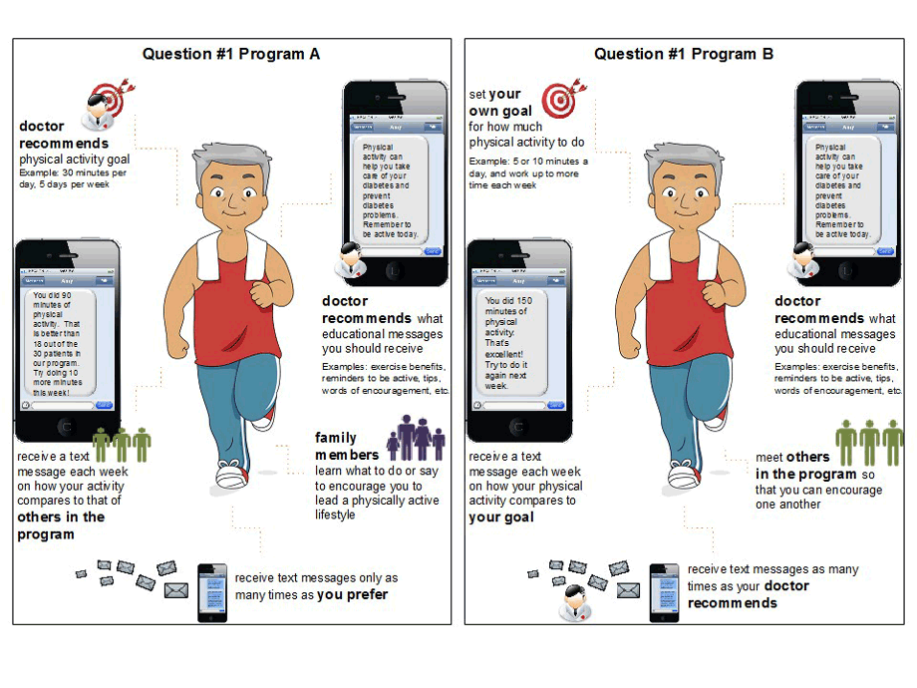
Two cards used for the first survey question in a discrete-choice experiment conducted to determine preferences for a text-messaging intervention designed to increase physical activity among low-income Latino men and women with diabetes in Los Angeles, California, 2014–2015. The top left sections of the cards describe the attribute of physical activity goal setting. The level for Program A is 1 (patient’s doctor recommends physical activity goals). For Program B, the level is 2 (patient selects his or her own personalized physical activity goals). The top right sections describe the attribute of physical activity behavior-change education, with level 1 (patient’s doctor recommends the educational content) assigned to both Program A and Program B. Three additional sections similarly depict the other 3 attributes.

### Step 3: Conduct survey to measure preferences

Using Orme’s calculation (based on 7 questions, 2 alternatives per question, and 2 levels per attribute), we determined that the minimum sample size to estimate a main-effects model was 71 (22); therefore, we set our goal as 125 respondents.

Any adult patient in the ambulatory care clinic’s diabetes management program, which serves approximately 1,200 patients per year, was eligible to participate in the survey. After a routine clinic visit, a patient was informed by a clinician that he or she was eligible to participate in a survey. If patients were interested, they were referred to the research assistant. Clinicians did not keep track of how many patients declined to participate. Recruitment took place from December 2014 to August 2015. All 125 patients who contacted the research assistant completed the survey. Patients signed a consent form and received $10.

The research assistant administered the survey in person at the clinic. For each of the 7 choice questions, the research assistant placed 2 cards in front of the respondent and described each alternative. The research assistant then asked the respondent, “If you were going to join one of these two programs to help you improve your physical activity, which one would you prefer?” The research assistant recorded the 7 responses for each respondent using a paper-based log.

### Step 4: Analyze the data

Methods to analyze DCEs put forth by Ryan et al (14) and Kuhfeld (21) guided our analysis. For each choice question, we assumed that the respondent would choose the alternative that led to higher utility (ie, value). Thus in a choice set consisting of 2 program alternatives, *i* and *j*, a respondent would choose program *j* if *U_j_* (*z_j_*, *c*) > *U_i_* (*z_i_*, *c*) in which *U* represents the respondent’s latent utility, *z* represents the attribute levels describing the alternative, and *c* represents the respondent’s characteristics. The latent utility is *U* = *V* + ε, in which *V* = *f *(*z*, *c*) is the deterministic component of utility and ε is the random component.

The choice model is the difference in utilities between program alternatives *i* and *j*. Because we observed choice rather than differences in utilities, we used a binary variable, *y_n_*, to reflect the *n^th^* respondent’s choice of program. Therefore, the form of the choice model is *y_n_* = (α + β*z_i_* + ∂*c_n_* + ε*_in_*) − (α + β*z_j_* + ∂*c_n_* + ε*_jn_*), in which α is the constant term, β represents the part-worth utility (the relative contribution of the attribute level to the overall utility derived from a particular alternative) of each attribute level, and ∂ represents the influence of respondent characteristics on choice of program. This choice model simplifies to *y_n_* = β (*z_i_* − *z_j_*) + (ε*_in_* − ε*_jn_*). That is, a respondent’s choice of program is a function only of the programs’ characteristics. We estimated the model using conditional logistic regression in SAS software. The coefficient estimates indicated (by statistical significance) whether the corresponding attributes were important to patients when they made decisions about their preferred TMI. The coefficient estimates also indicated (by relative size) how important each attribute was in relation to others. A positive coefficient for a given attribute indicated that a respondent preferred level 1 to level 2.

We can also assume that β (ie, the part-worth utility of each attribute level) depends on *c_n_*; that is, β = π + λ*c_n_*, in which π is a constant term and λ represents the influence of respondent characteristics on part-worth utility. The choice model thus becomes *y_n_* = [α + (π + λ*c_n_*)*z_i_* + ∂*c_n_* + ε*_in_*] − [α + (π + λ*c_n_*)*z_j_* + ∂*c_n_* + ε*_jn_*], which simplifies to *y_n_* = π(*z_i_* − *z_j_*) + λ*c_n_* (*z_i_* − *z_j_*) + (ε*_in_* − ε*_jn_*). We estimated this model using conditional logistic regression in SAS software to examine how preferences varied according to a respondent’s age, sex, and education. A significant coefficient indicated that attribute preferences varied by respondent characteristics.

To assess the models’ goodness of fit, we used χ^2^ tests, which test the null hypothesis that the independent variables do not influence choice. All *P *values less than .05 were considered significant.

## Results

The average age of the survey respondents was 52.6 years (Table 2). Most respondents were Latino (99.2%), female (71.2%), and preferred to speak Spanish (85.6%), and had less than a high school diploma (71.5%). The average number of years since receiving a diagnosis of diabetes was 10.8 years.

**Table 2:** Characteristics of Survey Respondents in a Survey in a Discrete-Choice Experiment Conducted to Determine Preferences for a Text-Messaging Intervention Designed to Increase Physical Activity Among Low-Income Latino Adults With Diabetes in Los Angeles, California, 2014–2015

Characteristic	No. of Respondents^a^	Value^b^
**Latino**	125	124 (99.2)
**Age, mean (SD), y**	124	52.6 (10.0)
**Female**	125	89 (71.2)
**Spanish is preferred language**	125	107 (85.6)
**Educational attainment**		
<High school diploma	123	88 (71.5)
High school graduate		22 (17.9)
>High school		13 (10.6)
**Annual household income**		
<$20,000	90	70 (56.0)
$20,000–$29,999		15 (12.0)
$30,000–$39,999		5 (4.0)
**Self-reported no. of years since diabetes diagnosis, mean (SD)**	125	10.8 (9.0)
**Level of comfort using text messaging**		
Very uncomfortable, uncomfortable, or neutral	124	40 (32.3)
Comfortable or very comfortable		84 (67.7)

Abbreviations: SD, standard deviation.

a Some respondents did not answer question.

b All values are number (percentage) unless otherwise indicated.

According to the main-effects–only model, 2 attributes significantly influenced preferences: frequency of messaging and physical activity behavior-change education. Respondents derived a greater utility from frequency of messaging (coefficient, 0.37; standard error [SE], 0.08; *P* < .001) than they did from physical activity behavior-change education (coefficient, 0.28; SE, 0.08; *P* < .001). Although the other attributes were not significant, social support had the next highest part-worth utility (coefficient, 0.10; SE, 0.08; *P* = .21), followed by feedback on physical activity performance (coefficient, 0.08, SE, 0.08; *P* = .32) and goal setting (coefficient, 0.06; SE, 0.08; *P* = .47).

Respondents preferred to have clinicians (rather than the patients themselves) recommend both frequency of messaging and content of the physical activity behavior-change education. Although the other attributes in the analysis of preferred levels were not significant, respondents preferred programs in which their families learn how to provide support (rather than programs in which patients support one another), feedback is based on individual physical activity performance (rather than feedback based on comparisons with other patients), and clinicians (rather than the patients themselves) recommend physical activity goals.

We found only one significant interaction in the analysis of the influence of patient characteristics on preferences: respondents with less than a high school diploma derived a greater utility from clinician-recommended physical activity goals than patients with a high school diploma or higher. Those with at least a high school diploma derived a greater utility from selecting their own individualized physical activity goals.

## Discussion

We illustrated how a DCE could be used to determine which TMI attributes are important to urban, low-income Latino patients with diabetes, the relative importance of the attributes, the preferred attribute levels, and how preferences vary by patient characteristics.

Despite the advantages of DCEs over traditional surveys and focus groups for eliciting information on preferences for health self-management technologies, this approach and the results it generates have limitations. The stated preferences of survey respondents for features of health self-management technologies may not align with the features they would choose in real-life settings (14). Revealed preference methods, on the other hand, address this limitation by measuring people’s preferences retrospectively through their choices (23). Revealed preference methods, however, would require data on actual health self-management technology choices, which to our knowledge are not available. Future research should assess the predictive value of DCEs by, for example, asking patients about their preferences for health self-management technologies and subsequently offering them a health self-management technology tool to see if they behave in accordance with what they stated in the DCE survey (24).

Although DCEs can distill features of health self-management technologies that are important to patients, there is no guarantee that technology incorporating patients’ preferred features would improve health outcomes. The technology would, however, have a higher likelihood of being accepted (and thus used) by patients (9). In a DCE, researchers select the features (ie, attributes) describing the hypothetical health self-management technologies that patients are asked to evaluate in a survey. The degree to which a technology designed in accordance with the preferred features will be effective depends on the features used in the DCE and how they were selected. As this study demonstrated, researchers should carefully select features based on a theoretical framework or empirical evidence or both. After preferred features are identified through a DCE, research should examine their impact on health outcomes.

A limitation of this study is that we used a convenience sample, so the findings should be interpreted with caution. We conducted this study primarily to demonstrate the use of DCEs to estimate, from the patient perspective, the importance of TMI features to support physical activity behavior change. Because we used a convenience sample, the results may not be generalizable to the broader population of urban, low-income adults with diabetes. However, based on a previous large-scale research study of patients in a diabetes management program at Los Angeles County Department of Health Services (25), we determined that our 125 study participants had similar characteristics with the target population of urban, low-income, predominantly Latino adults with diabetes.

Using a DCE to systematically quantify patient preferences for features of health self-management technologies is a feasible approach. However, discrepancies may exist between patients’ stated preferences and their actual behavior. Future research is needed to assess the extent to which DCEs predict patients’ choices of health self-management technologies in real-life settings. Moreover, we do not know whether technologies that address patient preferences improve health outcomes. Future research should investigate the impact of preferred technology features on health outcomes.

## Appendix Seven Choice Sets Used in a Survey in a Discrete-Choice Experiment Conducted to Determine Preferences for a Text-Messaging Intervention Designed to Increase Physical Activity Among Low-Income Latino Adults With Diabetes in Los Angeles, California, 2014–2015

**Table:**

Choice Set	Program Alternative	Attribute Level				
		Physical Activity Goal Setting	Feedback on Physical Activity Performance	Physical Activity Behavior-Change Education	Social Support	Frequency of Messaging
1	A	1	2	1	1	2
	B	2	1	1	2	1
2	A	1	1	2	1	2
	B	2	2	1	1	1
3	A	2	2	2	1	1
	B	1	2	1	2	2
4	A	1	2	1	2	2
	B	1	1	2	1	1
5	A	1	1	1	2	1
	B	2	2	2	2	2
6	A	2	1	2	2	2
	B	1	2	1	1	2
7	A	1	2	2	2	1
	B	2	1	1	1	2

## References

[1] Chen R , Cheadle A , Johnson D , Duran B . US trends in receipt of appropriate diabetes clinical and self-care from 2001 to 2010 and racial/ethnic disparities in care. Diabetes Educ 2014;40(6):756–66. 10.1177/0145721714546721 25142006

[2] Schillinger D , Bindman A , Wang F , Stewart A , Piette J . Functional health literacy and the quality of physician–patient communication among diabetes patients. Patient Educ Couns 2004;52(3):315–23. 10.1016/S0738-3991(03)00107-1 14998602

[3] Escarce J , Kapur K . Access to and quality of health care. In: Tienda M, Mitchell F, editors. Hispanics and the future of America. Washington (DC): National Academy Press; 2006. p. 410–46. 20669436

[4] Lopez-Quintero C , Berry EM , Neumark Y . Limited English proficiency is a barrier to receipt of advice about physical activity and diet among Hispanics with chronic diseases in the United States. J Am Diet Assoc 2009;109(10):1769–74. 10.1016/j.jada.2009.07.003 19782177

[5] Juckett G . Caring for Latino patients. Am Fam Physician 2013;87(1):48–54. 23317025

[6] Saffari M , Ghanizadeh G , Koenig HG . Health education via mobile text messaging for glycemic control in adults with type 2 diabetes: a systematic review and meta-analysis. Prim Care Diabetes 2014;8(4):275–85. 10.1016/j.pcd.2014.03.004 24793589

[7] López L , Grant RW . Closing the gap: eliminating health care disparities among Latinos with diabetes using health information technology tools and patient navigators. J Diabetes Sci Technol 2012;6(1):169–76. 10.1177/193229681200600121 22401336 PMC3320835

[8] US Department of Health and Human Services. Using health text messages to improve consumer health knowledge, behaviors, and outcomes: an environmental scan. 2014. http://www.hrsa.gov/healthit/txt4tots/environmentalscan.pdf. Accessed April 3, 2014.

[9] Or CK , Karsh BT , Severtson DJ , Burke LJ , Brown RL , Brennan PF . Factors affecting home care patients’ acceptance of a web-based interactive self-management technology. J Am Med Inform Assoc 2011;18(1):51–9. 10.1136/jamia.2010.007336 21131605 PMC3005875

[10] Sarkar U , Piette JD , Gonzales R , Lessler D , Chew LD , Reilly B , Preferences for self-management support: findings from a survey of diabetes patients in safety-net health systems. Patient Educ Couns 2008;70(1):102–10. 10.1016/j.pec.2007.09.008 17997264 PMC2745943

[11] Belmon LS , Middelweerd A , Te Velde SJ , Brug J . Dutch young adults ratings of behavior change techniques applied in mobile phone apps to promote physical activity: a cross-sectional survey. JMIR Mhealth Uhealth 2015;3(4):e103. 10.2196/mhealth.4383 26563744 PMC4704888

[12] Patrick K , Raab F , Adams MA , Dillon L , Zabinski M , Rock CL , A text message-based intervention for weight loss: randomized controlled trial. J Med Internet Res 2009;11(1):e1. 10.2196/jmir.1100 19141433 PMC2729073

[13] Sutherland J , Canwell D . Key concepts in marketing. New York (NY): Palgrave Macmillan; 2004. 84 p.

[14] Ryan M , Gerard K , Amaya-Amaya M , editors. Using discrete choice experiments to value health and health care. Dordrecht (NL): Springer Science and Business Media; 2007.

[15] Mühlbacher AC , Bethge S , Reed SD , Schulman KA . Patient preferences for features of health care delivery systems: a discrete choice experiment. Health Serv Res 2016;51(2):704–27. 10.1111/1475-6773.12345 26255998 PMC4799904

[16] Umpierre D , Ribeiro PA , Kramer CK , Leitão CB , Zucatti AT , Azevedo MJ . Physical activity advice only or structured exercise training and association with HbA1c levels in type 2 diabetes: a systematic review and meta-analysis. JAMA 2011;305(17):1790–9. 10.1001/jama.2011.576 21540423

[17] Nelson KM , Reiber G , Boyko EJ ; NHANES III. Diet and exercise among adults with type 2 diabetes: findings from the third National Health and Nutrition Examination Survey (NHANES III). Diabetes Care 2002;25(10):1722–8. 10.2337/diacare.25.10.1722 12351468

[18] Hu J , Amirehsani K , Wallace DC , Letvak S . Perceptions of barriers in managing diabetes: perspectives of Hispanic immigrant patients and family members. Diabetes Educ 2013;39(4):494–503. 10.1177/0145721713486200 23640301 PMC4054933

[19] Abraham C , Michie S . A taxonomy of behavior change techniques used in interventions. Health Psychol 2008;27(3):379–87. 10.1037/0278-6133.27.3.379 18624603

[20] Orme B . Formulating attributes and levels in conjoint analysis. Sequim (WA): Sawtooth Software; 2002.

[21] Kuhfeld W . Marketing research methods in SAS. Cary (NC): SAS Institute; 2005.

[22] Orme B . Sample size issues for conjoint analysis. Getting started with conjoint analysis: strategies for product design and pricing research. Madison (WI): Research Publishers Inc; 2010. p. 57–66.

[23] Samuelson PA . Consumption theory in terms of revealed preference. Economica 1948;15(60):243–53. 10.2307/2549561

[24] Ryan M , Watson V . Comparing welfare estimates from payment card contingent valuation and discrete choice experiments. Health Econ 2009;18(4):389–401. 10.1002/hec.1364 18677721

[25] Wu S , Ell K , Gross-Schulman SG , Sklaroff LM , Katon WJ , Nezu AM , Technology-facilitated depression care management among predominantly Latino diabetes patients within a public safety net care system: comparative effectiveness trial design. Contemp Clin Trials 2014;37(2):342–54. 10.1016/j.cct.2013.11.002 24215775

